# A natural human cell-adapted dengue type 3 virus strain

**DOI:** 10.1128/spectrum.00701-24

**Published:** 2024-06-07

**Authors:** Wy Ching Ng, Lowell Lin, Tanamas Siriphanitchakorn, Margaret Ke Xin Jiang, Hwee Cheng Tan, Eng Eong Ooi

**Affiliations:** 1 Programme in Emerging Infectious Diseases, Duke-NUS Medical School, Singapore, Singapore; 2 Viral Research and Experimental Medicine Centre, SingHealth Duke-NUS Academic Medical Centre, Singapore, Singapore; 3 Department of Clinical Translational Research, Singapore General Hospital, Singapore, Singapore; 4 Saw Swee Hock School of Public Health, National University of Singapore, Singapore, Singapore; National Taiwan University, Taipei, Taiwan

**Keywords:** dengue virus, dengue virus type 3, vaccine development

## LETTER

Dengue is an acute and occasionally life-threatening mosquito-borne disease. It is caused by infection with any one of four genetically distinct dengue viruses (DENV-1 to -4). Despite reports of each of the four DENVs showing differences in clinical outcome of infection, majority of pathogenesis studies have used DENV-1 and -2 (1). DENV-4 has received less focus due to its association with milder disease outcome (2). DENV-3, however, has caused large outbreaks and is associated with symptomatic infection and severe dengue (2). Its neglect in research relative to DENV-1 and -2 is, at least in part, due to difficulty in growing this virus, particularly in primary human cells to reasonable titers for pathogenesis studies. Difficulty in growing DENV-3 in approved mammalian cell lines (3) has also led to difficulties in supplying attenuated DENV-3 to complete the tetravalent formulation of dengue vaccines and even vaccine candidates. Indeed, low yields of attenuated DENV-3 cultures would reduce vaccine supply and increase cost of goods.

**Fig 1:**
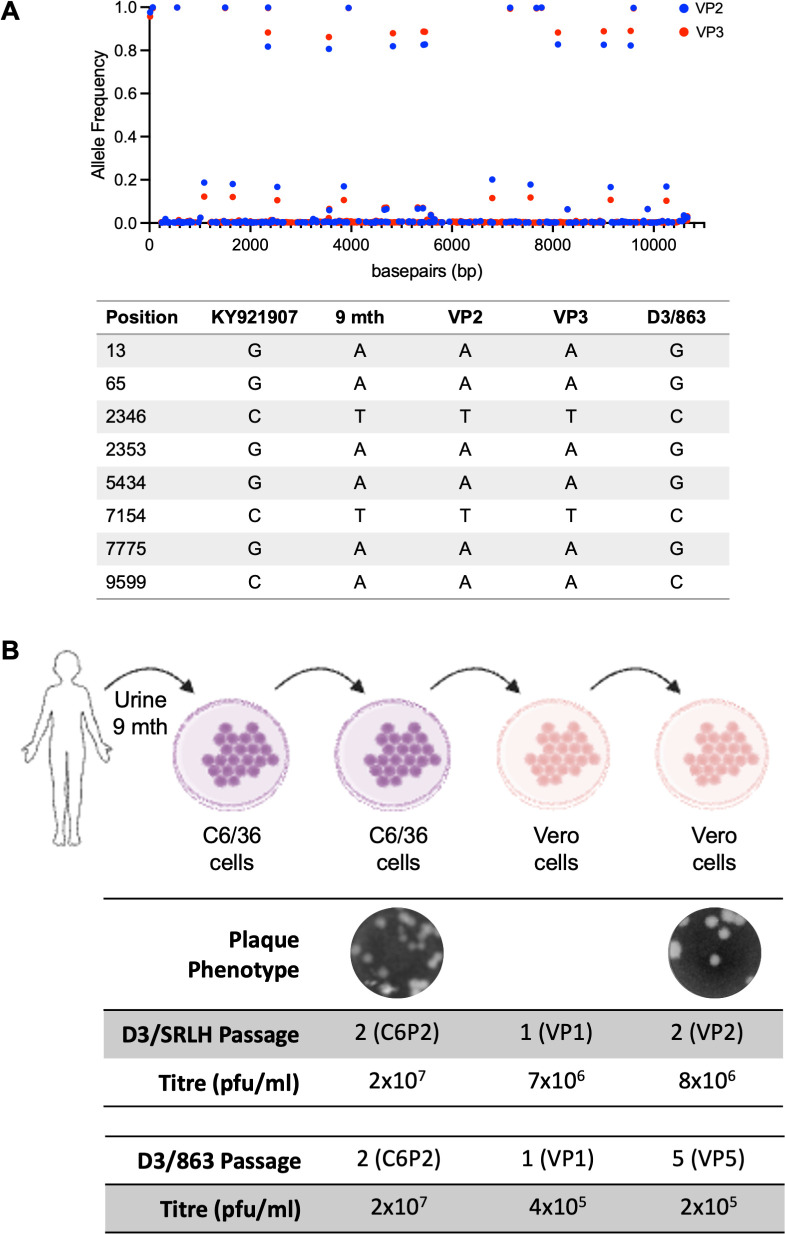
Isolation of high-titer D3/TZK virus. (**A**) Viral genome of D3/TZK is stable across passages with no new nucleotide changes after 2 and 3 passages in Vero cells. BLAST of consensus sequence showed the nearest match of D3/TZK to KY921907, a DENV3 SG(EHI)D3/15095Y15, which was isolated through the dengue surveillance system in the same period in Singapore as our patient. (**B**) D3/TZK was isolated from urine at 9 months of infection followed by two passages in C6/36 cells and further passages in Vero cells. D3/TZK virus amplified in both C6/36 and Vero cells to high titers.

Here, we suggest that a DENV-3 clinical isolate, DENV3/TZK/2016 (D3/TZK), obtained from an immunosuppressed allograft renal transplant recipient who developed dengue but who showed delayed infection clearance due to T cell lymphopenia (4), could be a useful strain for research. D3/TZK was isolated in C6/36 cells from urine samples taken at 4, 7, and 9 months after illness onset. Full genome sequencing of the virus at those three time points had revealed multiple mutations throughout the genome; more mutations were found in the isolate obtained at 9 months compared with that obtained at 4 months post-illness onset as well as to a contemporaneous DENV-3 isolate (Fig. 1A). These findings thus suggest that the 9-month D3/TZK could have undergone the greatest adaptation to human host (4). We thus further expanded the 9-month D3/TZK isolate *in vitro* and found that it was cultivable in both C6/36 and Vero cells to produce plaque titers of 2 × 10^7^ PFU/mL and 8 × 10^6^ PFU/mL, respectively; Vero cell expansion of a control virus, DENV-3 strain 863 (D3/863), a clinical isolate from an uncomplicated dengue patient in Singapore (5), produced 10-fold lower plaque titers (Fig. 1B). Critically, no change in plaque phenotype and no additional nucleotide polymorphism were detected in *in vitro*-expanded D3/TZK (Fig. 1B).

**Fig 2:**
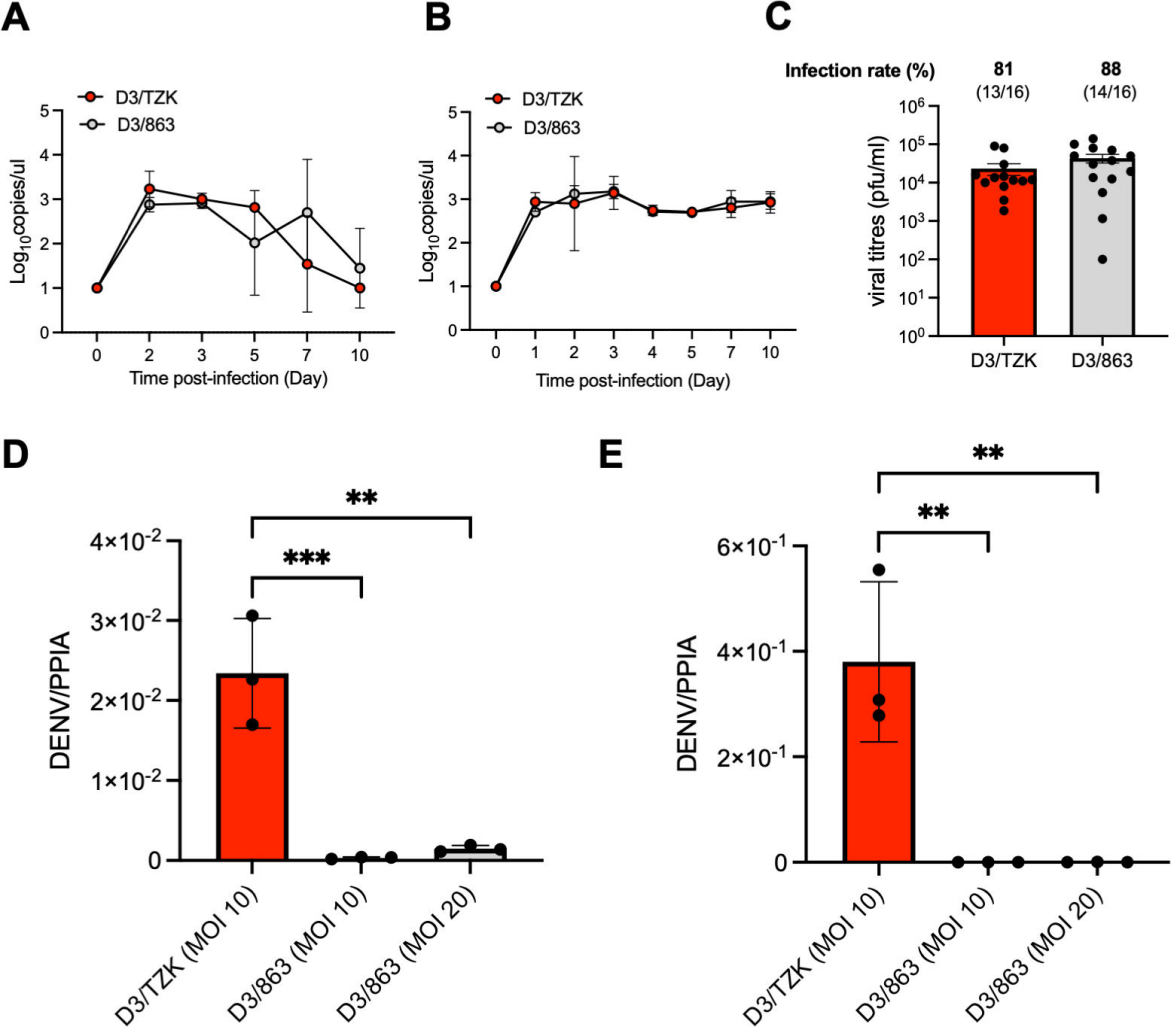
D3/TZK replicates in mammalian cells with uncompromised replication in mosquito and mice. (A and B) Viral RNA copies in serum of A129 (A) and AG129 (B) mice over time after infection with D3/TZK and D3/863. (C) Plaque titers of D3/TZK and D3/863 in whole *Aedes aegypti* at 14 days post-blood meal. (D and E) Increased DENV RNA genome 48 hours post infection in D3/TZK-infected monocytes (D) and moDCs (E). PPIA was used as the housekeeping gene for normalization.

For D3/TZK to be a useful research tool, it should infect and replicate to reasonable titers in experimental systems. We found that D3/TZK infection in type-I (A129) as well as type I and II interferon receptor-deficient (AG129) mice, both popularly used mouse models of dengue, produced viremia levels comparable with D3/863 (Fig. 2A and B). The two viruses also produced similar viral titers in *Aedes aegypti*, infected through a virus-spiked blood meal (Fig. 2C). Critically, however, D3/TZK showed productive infection in both primary human monocytes and monocyte-derived dendritic cells (moDCs) (Fig. 2D and E); inoculation of D3/863 onto these primary cells, even at a maximal MOI of 20, did not produce appreciable infection.

The unusually long duration of infection from suppressed cellular immunity in our patient may have allowed D3/TZK to be well adapted to human cells. Although there are unique mutations in its genome compared with other published DENV-3 genomes, D3/TZK could serve as a human cell-adapted prototype for research and vaccine development applications.
